# Einfluss des Behandlungssettings auf die Versorgung von Patient*innen mit rheumatoider Arthritis oder Psoriasisarthritis im Rahmen der Delegation an rheumatologische Fachassistenz — eine Post-hoc-Analyse der StärkeR-Studie

**DOI:** 10.1007/s00393-024-01606-8

**Published:** 2024-12-09

**Authors:** Rilind Shabani, Anna Mai, Robin Denz, Nina Timmesfeld, Jürgen Braun, Dietmar Krause

**Affiliations:** 1 Abteilung für medizinische Informatik, Biometrie und Epidemiologie, Ruhr-Universität Bochum, 44780 Bochum, Deutschland; 2 Rheumatologisches Versorgungszentrum Steglitz, Berlin, Deutschland

**Keywords:** Rheumatoide Arthritis, Patientenversorgung, Versorgungsqualität, Klinikambulanz, Praxis, Rheumatoid arthritis, Patient care, Quality of care, Hospital outpatient department, Practice

## Abstract

**Hintergrund:**

Die StärkeR-Studie zeigte die Nichtunterlegenheit einer teambasierten Versorgungsform mit Delegation an rheumatologische Fachassistenz (RFA) gegenüber der Standardversorgung bei Patient*innen mit rheumatoider Arthritis (RA) oder Psoriasisarthritis (PsA).

**Zielsetzung:**

Explorative Analysen hinsichtlich eines möglichen Einflusses des Behandlungssettings (Schwerpunktpraxis/Klinikambulanz) auf verschiedene Outcome-Parameter bei Patient*innen mit RA oder PsA im Rahmen der Delegation an rheumatologische Fachassistenz.

**Material und Methoden:**

Patient*innen mit RA oder PsA und stabiler Einstellung bei geringer Krankheitsaktivität aus 3 Klinikambulanzen und 14 rheumatologischen Schwerpunktpraxen, die an der StärkeR-Studie teilgenommen hatten, wurden in diese Post-hoc-Analyse einbezogen. Die Effektivität der teambasierten Versorgungsform in Abhängigkeit vom Behandlungssetting wurde mittels Interaktionsanalysen in linearen Regressionsmodellen u. a. mit Blick auf Krankheitsaktivität, Funktionsfähigkeit, Schmerz und Müdigkeit untersucht.

**Ergebnisse:**

Von 588 Patient*innen wurden 466 in Schwerpunktpraxen behandelt, 92 in Klinikambulanzen. Die Analysen ergaben bei einem von 9 untersuchten Endpunkten eine signifikante Interaktion: Die Funktionskapazität (Skala 0–1) zeigte in den Klinikambulanzen gegenüber der Standardversorgung gering niedrigere Werte (−0,07 [−0.12; −0.02]), während sich ein solcher Unterschied in den Praxen nicht darstellte. Bei anderen Endpunkten zeigte sich tendenziell ein Vorteil der teambasierten Versorgungsform im Praxissetting.

**Diskussion:**

Diese explorativen Analysen weisen auf den möglichen Nutzen der Evaluation von verschiedenen Versorgungsformen wie der Delegation ärztlicher Leistungen an qualifizierte RFA im Sinne eines Benchmarkings hin.

## Abstract (en)

**Background:**

The StärkeR study has shown the non-inferiority of a team-based form of care with delegation to rheumatological specialist assistants (RFA) compared to standard care in patients with rheumatoid arthritis (RA) or psoriatic arthritis (PsA).

**Objective:**

Exploratory analyses regarding a possible influence of the treatment setting (specialist practice/outpatient clinic) on various outcome parameters in patients with RA or PsA in the context of delegation to RFA.

**Material and methods:**

Patients with RA or PsA and stable adjustment with low disease activity from 3 outpatient clinics and 14 rheumatological specialist practices that participated in the StärkeR study were included in this post hoc analysis. The effectiveness of the team-based form of care depending on the treatment setting was investigated using interaction analyses in linear regression models with respect to disease activity, functional capacity, pain and fatigue, among others.

**Results:**

Out of 588 patients 466 were treated in specialized practices and 92 in hospital outpatient clinics. The analyses showed a significant interaction for one of nine outcomes examined: functional capacity (scale 0–1) had slightly lower values in the hospital outpatient clinics compared to standard care (−0.07 [−0.12; −0.02]), while no such difference was found in the practices. For other outcomes, the team-based form of care in the practice setting tended to show an advantage.

**Discussion:**

These exploratory analyses point to the potential benefits of evaluating different forms of care, such as the delegation of medical services to qualified RFA, in terms of benchmarking.

Die Versorgung von rheumatologischen Erkrankungen wie der rheumatoiden Arthritis (RA) oder der Psoriasisarthritis (PsA) ist in einigen Regionen unzureichend [1]. Es gibt Empfehlungen seitens der EULAR (European Alliance of Associations for Rheumatology), durch Delegation an speziell ausgebildetes nichtärztliches Fachpersonal (teambasierte Versorgungsform) eine verbesserte Versorgung für Rheuma-Patient*innen zu erreichen [2]. Internationale und auch deutsche Studien hatten bereits Vorteile einer teambasierten Versorgung gegenüber der Standardversorgung zeigen können [3].

## Hintergrund und Fragestellung

Die vom Innovationsfonds geförderte randomisierte Studie „Strukturierte Delegation ärztlicher Leistungen im Rahmen konzeptionsgeregelter Kooperation in der Versorgung von Patient*innen mit entzündlichem Rheuma (StärkeR)“ hatte sich zum Ziel gesetzt, die teambasierte Versorgungsform mit Delegation an rheumatologische Fachassistenz (RFA) im Hinblick auf die Effektivität hinsichtlich der Krankheitsaktivität und der gesundheitsbezogenen Lebensqualität im deutschen Gesundheitssystem zu untersuchen (Studienprotokoll zur StärkeR-Studie [4]). Die teambasierte Versorgungsform erwies sich hinsichtlich der Veränderung der Krankheitsaktivität (gemessen mit dem DAS28 [BSG]) nach einem Jahr als der Standardversorgung nicht unterlegen [5]. Ausgehend von Befragungen unter den beteiligten Ärzt*innen und rheumatologischen Fachassistentinnen (RFA) sowie in Fokusgruppen mit RFA, die zum Ende des StärkeR-Projektes durchgeführt wurden, zeigten sich Unterschiede in der Umsetzung bzw. Umsetzbarkeit des Delegationskonzeptes zwischen rheumatologischen Schwerpunktpraxen und Klinikambulanzen [6].

Internationale Studien geben bereits Hinweise, dass das Behandlungssetting Einfluss auf das Behandlungsergebnis bei teambasierter Versorgung haben kann [7, 8]. Bisher existiert jedoch keine einheitliche Erkenntnis darüber, ob eine Versorgung mit Delegation in Klinikambulanzen und Praxen gleichermaßen funktioniert [9]. Zudem ist die Übertragung der Ergebnisse internationaler Studien auf Deutschland aufgrund unterschiedlicher Gesundheitssysteme problematisch. Für das deutsche Gesundheitssystem gibt es bislang keine Studie, die die Effektivität der Delegation in den Behandlungssettings Klinikambulanz und Praxis vergleichend analysiert. Es ist jedoch bekannt, dass es grundsätzlich Unterschiede in den Strukturen und dem therapeutischen Vorgehen in der Behandlung von Patient*innen zwischen Praxen und Kliniken gibt [6]. Anhand von Post-hoc-Auswertungen der StärkeR-Daten untersuchten wir daher den Einfluss des Behandlungssettings (Klinikambulanz vs. Schwerpunktpraxis) auf den Behandlungserfolg der teambasierten Versorgung mit Delegation im Vergleich zur Standardversorgung von Patient*innen mit RA oder PsA.

## Methodik

Im Rahmen der StärkeR-Studie wurden zwischen September 2018 und Juni 2019 insgesamt 601 stabil eingestellte Patient*innen mit RA und PsA in 3 Klinikambulanzen und 14 rheumatologischen Schwerpunktpraxen aus Nordrhein-Westfalen und Niedersachsen in die Studie eingeschlossen. Die Datenerhebung erfolgte zu Baseline und im 3‑monatigen Rhythmus zuletzt nach einem Jahr.

### Explorative Post-hoc-Auswertungen

Betrachtete Endpunkte dieser Arbeit sind die Krankheitsaktivität (DAS28-BSG), Funktionskapazität (Funktionsfragebogen Hannover), Entzündungsparameter (log(CRP)) sowie patientenberichtete Parameter wie Lebensqualität (EuroQol‑5 Dimension‑5 Level [EQ-5D-5L]), Depression (Patient Health Questionnaire [PHQ-2-Score]), globale Einschätzung der Krankheitsaktivität, Schlaflosigkeit, Müdigkeit und Schmerz. Details zur Erhebung sind dem Studienprotokoll zur StärkeR-Studie zu entnehmen [4]. EQ-5D-5L ist ein Instrument zur Bewertung der gesundheitsbezogenen Lebensqualität. Es umfasst 5 Dimensionen (5-stufige Version): Mobilität, Selbstversorgung, gewohnte Aktivitäten, Schmerzen/Beschwerden und Angst/Depression, wobei in jeder Dimension 5 Schweregrade möglich sind: keine (Stufe 1), leichte (Stufe 2), mäßige (Stufe 3), schwere (Stufe 4) und extreme Probleme/Unfähigkeit (Stufe 5) [10]. Der PHQ-2-Score ist ein Kurzfragebogen zur Bewertung der depressiven Symptome bei Patient*innen. Diese wurden gebeten, auf einer 4‑stufigen Skala einzuschätzen, wie häufig sie sich in den vergangenen 2 Wochen durch Beschwerden beeinträchtigt fühlten. Die Skala reichte von „überhaupt nicht“ bis „beinahe jeden Tag“. Im PHQ-2-Score werden Fragen 1. zum Interesse und zur Freude an den ausgeübten Tätigkeiten sowie 2. zu Niedergeschlagenheit, Schwermut und Hoffnungslosigkeit bestellt. Der Score kann Werte von 0 bis 6 annehmen. Ein höherer Score deutet auf eine höhere Belastung durch depressive Symptome hin. Krankheitsaktivität, Müdigkeit, Schlaflosigkeit und Schmerz wurden auf einer numerischen Rating-Skala von 0 (gar nicht) bis 10 (sehr stark) erfasst [11].

Zunächst wurden Patientencharakteristika deskriptiv getrennt nach Behandlungsgruppe und Behandlungssetting dargestellt. Aufgrund der ungleichen Verteilung der CRP-Daten wurden der Median und der Interquartilsabstand (Q25–Q75) der Veränderungen angegeben und logarithmierte CRP-Werte in die Modelle (s. unten) eingefügt. Für metrische Variablen wurden ANOVA-Tests, für kategoriale Variablen Chi-Quadrat-Tests durchgeführt, um zu prüfen, ob es einen generellen Unterschied in der Verteilung der Variablen zwischen den Untergruppen gibt. Die *p*-Werte wurden nicht für multiples Testen adjustiert.

Im Anschluss wurden lineare Regressionsmodelle berechnet, um die Veränderungen des jeweiligen Endpunkts im Verlaufe eines Jahres zu untersuchen. Dabei stellten die Behandlungsgruppe (teambasiert oder Standardversorgung) und das Behandlungssetting (Praxis oder Klinikambulanz) die zentralen unabhängigen Variablen dar. Um den Unterschied des Behandlungseffekts je nach Behandlungssetting zu modellieren, wurde neben den Haupteffekten zusätzlich der Interaktionsterm dieser beiden Variablen berücksichtigt. Analog zur StärkeR-Hauptauswertung wurden außerdem stets der Baselinewert des jeweiligen Outcomes, das Geschlecht und die Art der Erkrankung (RA oder PsA) mit ins Modell aufgenommen. Formal haben die Modelle die folgende Form:

$$\begin{aligned}Y=&\beta _{0}+\beta _{1}\cdot \textit{Behandlungsgruppe}\\&+\beta _{2}\cdot \textit{Behandlungssetting}\\&+\beta _{3}\cdot (\textit{Behandlungsgruppe}\\&\quad\times \textit{Behandlungssetting})\\&+\beta _{4}\cdot \textit{Baselinewert}\\&+\beta _{5}\cdot \textit{Geschlecht}\\&+\beta _{6}\cdot \textit{Erkrankungsart}+\epsilon\end{aligned}$$

Die Betas (β) repräsentieren die Regressionskoeffizienten bzw. die Effekte der jeweiligen unabhängigen Variablen auf Y, während $\epsilon$ den Fehlerterm darstellt.

Im Gegensatz zur StärkeR-Hauptauswertung wurde kein zufälliger Praxiseffekt berücksichtigt, da dieser sonst einen Teil des Effekts des Behandlungssettings tilgen würde. Die resultierenden Schätzungen [12] werden stets mit zugehörigen 95 %-Konfidenzintervallen und, da es sich um explorative Auswertungen handelt, unadjustierten *p*-Werten berichtet.

Alle Analysen wurden mit der Programmiersprache R (Version 4.2.2, 08.11.2023, The R Project for Statistical Computing, r-project.org) durchgeführt.

## Ergebnisse

### Die Patient*innen

**Abb. 1:**
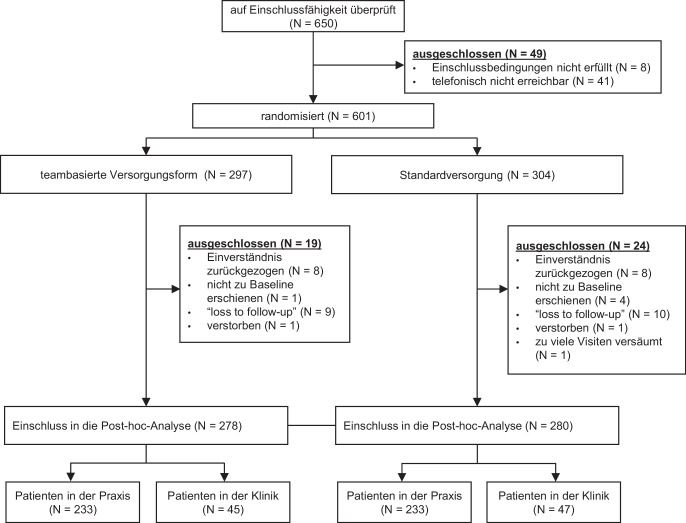
Flussdiagramm zum Projekt „Analysen zum Einfluss des Behandlungssettings auf die Effektivität einer neuen Versorgungsform“

Von den ursprünglich 601 in der StärkeR-Studie randomisierten Patient*innen mussten 19 bzw. 24 in der teambasierten Versorgung bzw. der Standardversorgung ausgeschlossen werden, da sie ihr Einverständnis zurückgezogen hatten, nicht zur ersten Studienvisite erschienen oder im weiteren Verlauf der Studie nicht mehr erreichbar waren. Letztendlich wurden 558 Patient*innen in die vorliegende Studie einbezogen. Das Verhältnis von Praxis- zu Klinikambulanzpatient*innen betrug in etwa 4:1, d. h. 466 Personen wurden im niedergelassenen Setting behandelt, 92 Personen in einer Klinikambulanz. Die Verteilung auf die Versorgungsformen (teambasierte und Standardversorgung) erfolgte durch Randomisierung (Abb. 1).

Die Charakteristika der Patient*innen, aufgeteilt auf die Behandlungsgruppen und Behandlungssettings, sind der Tab. 1 zu entnehmen. Über alle Gruppen hinweg waren die Patient*innen im Durchschnitt 63 Jahre alt, der Anteil an Frauen lag bei 78 %. In den Subgruppen deuten sich jedoch Unterschiede an. So waren Patient*innen der Klinikambulanzen tendenziell jünger und depressiver als jene, die in Praxen behandelt wurden; sie gaben stärkere Müdigkeit und größere Schlafstörungen an. Außerdem zeigten sich trotz Randomisierung tendenziell Unterschiede zwischen den Behandlungsgruppen im Kliniksetting. Jene, die teambasiert versorgt wurden, waren häufiger männlich (31 % vs. 15 %), hatten eine höhere Krankheitsaktivität (gemessen am DAS28 und selbst berichtet), höhere CRP-Werte, geringere Funktionsfähigkeit sowie höhere Schmerzintensität als jene, die der Standardversorgung zugeteilt worden waren. In den Praxen wiesen umgekehrt die Patient*innen der Standardversorgung tendenziell schlechtere Werte im Vergleich zur teambasierten Versorgung auf.

**Tab. 1:** Baseline-Patientencharakteristika nach Subgruppen

	Praxis (*N* = 466)		Klinikambulanz (*N* = 92)		*p*-Wert	Alle (*N* = 558)
	Team (*N* = 233)	Standard (*N* = 233)	Team (*N* = 45)	Standard (*N* = 47)		
Frauen, *N (%)*	179 (77)	185 (79)	31 (69)	40 (85)	0,261	435 (78)
Alter (Jahre) *MW (SD)*	64 (11)	63 (12)	60 (15)	58 (11)	0,011	63 (12)
DAS28 (0–9,4) *MW (SD)*	3,05 (1,24)	3,19 (1,25)	3,11 (1,29)	2,81 (1,06)	0,235	3,09 (1,24)
EQ-5D-5L (≤1) *MW (SD)*	0,75 (0,24)	0,73 (0,25)	0,70 (0,25)	0,75 (0,25)	0,408	0,74 (0,25)
Funktionskapazität (FFbH, 0–1) *MW (SD)*	0,76 (0,21)	0,75 (0,22)	0,74 (0,26)	0,77 (0,22)	0,909	0,76 (0,22)
Krankheitsaktivität (NRS, 0–10) *MW (SD)*	3,4 (2,2)	3,8 (2,3)	4,1 (2,6)	3,3 (2,0)	0,137	3,6 (2,3)
Depression (PHQ‑2, 0–6) *MW (SD)*	1,3 (1,4)	1,4 (1,5)	1,9 (1,8)	1,8 (1,7)	0,384	1,5 (1,5)
CRP (mg/l) *Median (IQR)*	2,27 (1,31)	2,16 (1,29)	2,06 (1,67)	1,83 (1,12)	0,1	2,18 (1,29)
Schmerzintensität (NRS, 0–10) *MW (SD)*	3,5 (2,4)	3,9 (2,4)	4,6 (2,8)	3,4 (2,1)	0,026	3,7 (2,4)
Müdigkeit (NRS, 0–10) *MW (SD)*	3,8 (3,0)	3,8 (2,9)	4,7 (2,9)	4,1 (2,9)	0,332	3,9 (3,0)
Schlafstörungen (NRS, 0–10) *MW (SD)*	3,8 (3,1)	4,2 (3,1)	4,9 (2,9)	4,5 (3,4)	0,106	4,1 (3,1)

*MW* Mittelwert, *SD* Standardabweichung, *IQR* Interquartilsabstand, *Team* teambasierte Versorgungform, *DAS28* Disease Activity Score mit 28 Gelenken, *EQ-5D-5L* EuroQol in 5 Dimensionen und 5 Antwortstufen, *CRP* C-reaktives Protein (nicht logarithmiert),* FFbH* Funktionsfragebogen Hannover, *NRS* numerische Rating-Skala (0–10), *PHQ‑2* Patient Health Questionnaire 2 Items (0–6)

### Die Behandelnden

In dieser Post-hoc-Analyse waren Daten von 31 RFAs und 25 Rheumatolog*innen verfügbar. Die Ärzteschaft in den Klinikambulanzen war im Durchschnitt 49 ± 11 Jahre alt, in den Praxen im Mittel knapp 10 Jahre älter. Entsprechend hatten die Ärzt*innen in den Praxen im Mittel mehr Berufserfahrung in der Rheumatologie. Die Gruppen der RFA unterschieden sich in Klinik und Praxis mit Blick auf das mittlere Alter nur unwesentlich (Tab. 2). Während die teambasierte Versorgung ausschließlich von weiblichen RFA angeboten wurde, lag in der Standardversorgung der Anteil an Frauen unter den Niedergelassenen bei 27 %, unter der Klinikärzteschaft bei 40 %.

**Tab. 2:** Charakteristika der Behandelnden

	Praxis (*n* = 40)		Klinikambulanz (*n* = 16)		Alle (*n* = 56)
	RFA^a^ (*n* = 25) – teambasierte Versorgung	Ärzte (*n* = 15) – Standardversorgung	RFA^a^ (*n* = 6) – teambasierte Versorgung	Ärzte (*n* = 10) – Standardversorgung	
Alter (Jahre) *MW (SD)*	44 (12)	58 (4)	47 (11)	49 (11)	49 (11)
Frauen *n (%)*	25 (100)	4 (27)	6 (100)	4 (40)	39 (70)
Berufserfahrung in der Rheumatologie (Jahre) *MW (SD)*	10 (7)	24 (4)	15 (10)	16 (13)	15 (10)

*MW* Mittelwert, *SD* Standardabweichung

^a^Da in der teambasierten Versorgungsform die gleichen Ärztinnen und Ärzte beteiligt waren wie in der Standardversorgung, werden die Charakteristika der Behandler in der teambasierten Versorgungsform rein auf die RFA bezogen

### Veränderungen nach einem Jahr „treat to target“

In Tab. 3 sind zunächst die Veränderungen aller betrachteten Endpunkte nach einem Jahr teambasierter Versorgung mit Delegation an RFA oder Standardversorgung durch Ärzt*innen jeweils in den Klinikambulanzen und Praxen dargestellt. Insgesamt zeigen sich für das Praxissetting durchweg Verbesserungen über die Zeit in beiden Versorgungsformen, während das Bild im Kliniksetting uneinheitlich ist. Dabei sind die Veränderungen nach einem Jahr „treat to target“ in allen Gruppen und Endpunkten eher gering.

**Tab. 3:** Veränderungen nach einem Jahr „treat to target“ in Klinikambulanzen und Praxen nach Versorgungsform

	Praxis Geschätzte Veränderung^a^ [95 %-KI]				Klinikambulanz Geschätzte Veränderung^a^ [95 %-KI]			
	Team		Standard			Team		Standard
DAS28 (0–9,4)	−0,26	[−0,42; −0,1]	−0,05	[−0,21; 0,11]	−0,1	[−0,4; 0,21]	0,02	[−0,29; 0,32]
Log(CRP) (mg/l)	−0,15	[−0,2; −0,1]	−0,08	[−0,13; −0,03]	−0,09	[−0,18; 0,01]	−0,12	[−0,22; −0,02]
Krankheitsaktivität (NRS, 0–10)	−0,46	[−0,77; −0,15]	−0,2	[−0,52; 0,12]	0,22	[−0,39; 0,82]	−0,37	[−0,99; 0,26]
Schmerzintensität (NRS, 0–10)	−0,36	[−0,68; −0,04]	−0,09	[−0,41; 0,24]	0,03	[−0,6; 0,65]	0,08	[−0,55; 0,72]
Müdigkeit (NRS, 0–10)	−0,26	[−0,59; 0,08]	−0,04	[−0,39; 0,3]	0,18	[−0,47; 0,84]	−0,16	[−0,83; 0,5]
Schlafstörungen (NRS, 0–10)	−0,38	[−0,76; 0,01]	−0,04	[−0,43; 0,35]	−0,25	[−1; 0,51]	−0,47	[−1,23; 0,29]
Depression (PHQ‑2, 0–6)	−0,12	[−0,31; 0,08]	−0,11	[−0,3; 0,08]	−0,46	[−0,88; −0,05]	−0,22	[−0,61; 0,18]
EQ-5D-5L^b^ (≤ 1)	0,05	[0,02; 0,09]	0,03	[0; 0,06]	−0,02	[−0,08; 0,04]	−0,02	[−0,08; 0,04]
Funktionskapazität^b^ (FFbH, 0–1)	0,001	[−0,01; 0,02]	0,00	[−0,02; 0,02]	−0,07	[−0,11; −0,04]	0,00	[−0,04; 0,03]

*KI* Konfidenzintervall, *MW* Mittelwert, *SD* Standardabweichung, *IQR* Interquartilsabstand, *Team* teambasierte Versorgungform, *DAS28* Disease Activity Score mit 28 Gelenken und BSG, *EQ-5D-5L* EuroQol in 5 Dimensionen und 5 Antwortstufen, *CRP* C-reaktives Protein (nicht logarithmiert), *FFbH* Funktionsfragebogen Hannover, *NRS* numerische Rating-Skala (0–10), *PHQ‑2* Patient Health Questionnaire 2 Items (0–6)

^a^Endwert – Anfangswert

^b^Ein negatives Vorzeichen bedeutet eine Verschlechterung

### Einfluss des Behandlungssettings auf die Ergebnisse der teambasierten Versorgung

**Abb. 2:**
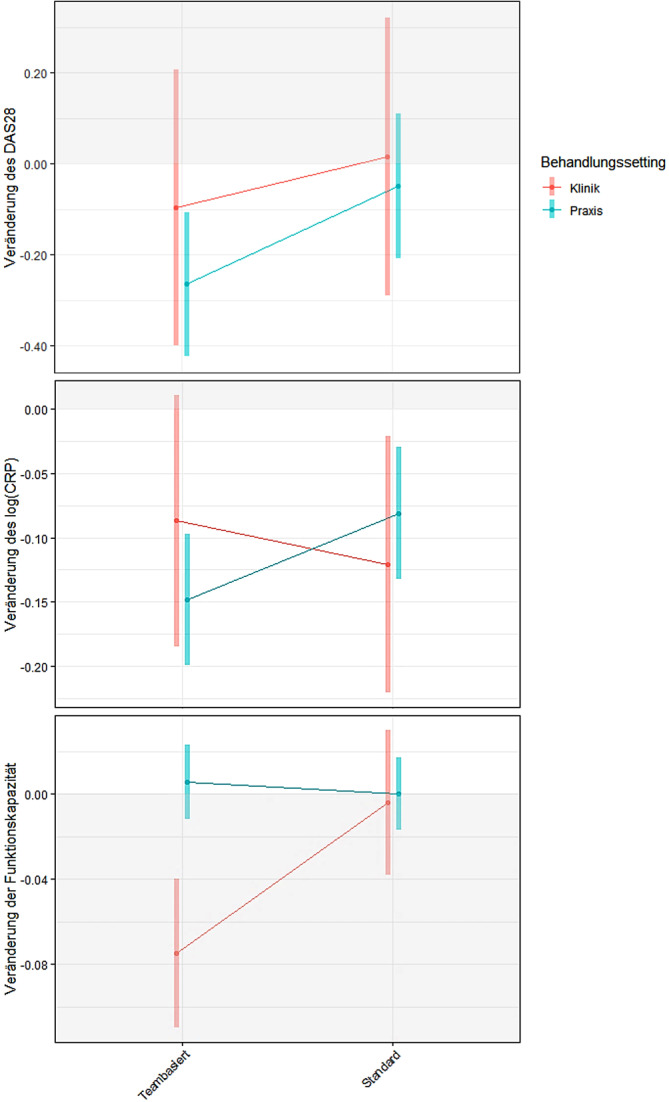
Veränderungen von Krankheitsaktivität (DAS28 [BSG]), CRP und Funktionskapazität (Funktionsfragebogen Hannover) nach einem Jahr „treat to target“ in Klinikambulanzen und Praxen nach Versorgungsform (*grau hinterlegt* ist der Bereich der Verschlechterung). *DAS28* Disease Activity Score mit 28 Gelenken und BSG, *log(CRP)* C-reaktives Protein (logarithmiert), *FFbH* Funktionsfragebogen Hannover

Die grafische Veranschaulichung ausgewählter Endpunkte (Abb. 2) gibt einen ersten Eindruck von einer möglichen Interaktion zwischen Behandlungsform und Behandlungssetting. Auf der x‑Achse stehen die Settings vergleichend nebeneinander, auf der y‑Achse ist die Veränderung des jeweiligen Endpunktes nach einem Jahr abgetragen; der weiße Bereich markiert den Bereich einer Verbesserung, der graue den einer Verschlechterung im Outcome nach einem Jahr. Verlaufen die Geraden parallel zueinander, so erfolgen die Veränderungen in den Settings auf einem unterschiedlichen Niveau, jedoch ohne dass eine Interaktion mit dem Setting vorliegt. Zeigt sich ein gegenläufiger Verlauf, so deutet dies auf eine unterschiedliche Wirksamkeit der Versorgungsform in Abhängigkeit vom Setting hin. Die Abb. 2 zeigt einige entgegengesetzte Verläufe, wobei die weiten Konfidenzintervalle, v. a. im Kliniksetting aufgrund der geringen Fallzahl, auf unsichere Schätzungen hindeuten.

**Abb. 3:**
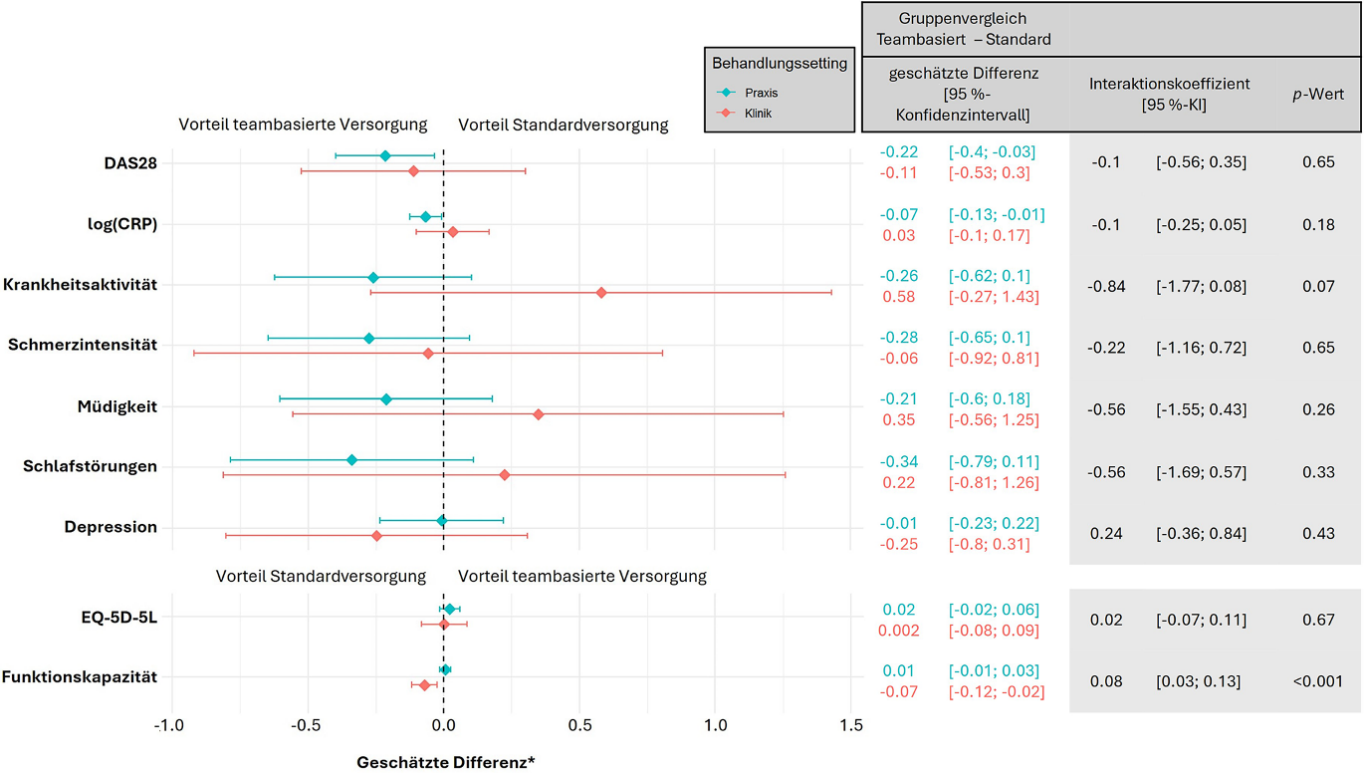
Effektivität der neuen Versorgungsform mit Delegation im Vergleich zur Standardversorgung in Abhängigkeit vom Behandlungssetting. *DAS28* Disease Activity Score mit 28 Gelenken und BSG, *log(CRP)* C-reaktives Protein (logarithmiert), *Krankheitsaktivität* globale Krankheitsaktivität auf numerischer Rating-Skala NRS (0–10), *Schmerzintensität, Müdigkeit, Schlafstörungen* auf NRS (0–10), *PHQ‑2* Patient Health Questionnaire 2 Items (0–6), *EQ-5D-5L* EuroQol in 5 Dimensionen und 5 Antwortstufen, *FFbH* Funktionsfragebogen *Hannover,* * geschätzt anhand der Modelle

Vergleicht man nun die Ergebnisse der teambasierten Versorgung mit der Standardversorgung, so zeigt sich in den Schwerpunktpraxen eine signifikante Überlegenheit der teambasierten Versorgungsform für den DAS28 (geschätzte Differenz teambasierte Versorgung – Standardversorgung [95 %-KI]: −0,22 [−0,40; −0,03]) und das logCRP (−0,07 [−0,13; −0,01]), allerdings ohne signifikante Interaktionen zwischen Behandlungsform und Behandlungssetting (s. Abb. 3). In der Klinik ergaben die explorativen Analysen beim Endpunkt Funktionsfähigkeit eine signifikante Unterlegenheit der teambasierten Versorgungsform (Skala 0–1: −0,07 [−0,12; −0,02]). Die multivariable lineare Regressionsanalyse ergab nach Adjustierung für den Baselinewert, das Geschlecht der Patient*innen und die Art der Vorerkrankung (PsA vs. RA) eine signifikante Interaktion, d. h. eine Abhängigkeit der Ergebnisse der teambasierten Versorgungsform vom Behandlungssetting. Für alle anderen Outcomes konnten keine bedeutsamen Unterschiede zwischen den Versorgungformen und zwischen den Settings nachgewiesen werden.

## Diskussion

Die vorliegenden explorativen Auswertungen deuten darauf hin, dass es Unterschiede in den Ergebnissen der teambasierten Versorgungsform zwischen den Behandlungssettings geben könnte. Es zeigte sich, dass die teambasierte Versorgungsform in den Praxen mit Blick auf krankheitsrelevante Endpunkte wie DAS28 und CRP signifikant effektiver war als die Standardversorgung. Hingegen stellte sich in den Klinikambulanzen dieser Vorteil der teambasierten Versorgung nicht dar. Vielmehr deutet sich durch die Richtung der geschätzten Differenzen anhand mehrerer Endpunkte tendenziell eher ein Nachteil der teambasierten Versorgungsform in den Klinikambulanzen an. Dies war insbesondere bei der Veränderung der Funktionskapazität der Patient*innen innerhalb eines Jahres der Fall. Hier ergab die Interaktionsanalyse einen signifikant unterschiedlichen Behandlungseffekt in Abhängigkeit vom Behandlungssetting. Die gefundenen Unterschiede sind aber eher gering und bedürfen zur Bestätigung sicherlich weiterer, insbesondere konfirmatorischer Untersuchungen über die teambasierte Versorgungsform in verschiedenen Behandlungssettings.

Vergleichende internationale Studien liegen zu dem Thema aktuell nicht vor. In einer Metaanalyse [13] zeigte sich, dass eine teambasierte Versorgungsform („nurse-led care“) in Bezug auf verschiedene klinische Endpunkte wie Krankheitsaktivität, Funktion und Lebensqualität der Patient*innen grundsätzlich ebenso gute Ergebnisse erzielt wie die Standardversorgung (konventionelles ärztliches Follow-up). Eine Studie aus Schweden [14] untersuchte eine teambasierte Versorgungsform im Kliniksetting und zeigte anders als diese Arbeit einen Trend zur Verbesserung der Outcomes in der Klinik. Vergleichbare Ergebnisse lieferte eine qualitative Analyse zur teambasierten Versorgungsform im Kliniksetting [15]. Jedoch untersuchten diese und viele weitere internationale Studien nur ein Behandlungssetting, sodass ein Vergleich der Effektivität einer teambasierten Versorgung zwischen den Settings nicht möglich ist. Die vorliegende Arbeit ist die erste Studie, die einen Einfluss des Behandlungssettings auf die Effektivität einer Versorgungsform mit Delegation mittels Interaktionsanalysen untersucht hat.

Für die Interpretation der hier dargestellten Ergebnisse müssen allerdings einige methodische Limitationen berücksichtigt werden. Die Auswertungen erfolgten explorativ im Nachgang an die Beantwortung der Hauptfragestellung der StärkeR-Studie. Entsprechend ist die fehlende Power für die Interaktionsanalysen dieser Arbeit zu nennen. Durch die kleinere Klinik-Kohorte ergaben sich unpräzisere Schätzungen, erkennbar an den weiten Konfidenzintervallen im Kliniksetting. Die Ergebnisse sollten daher zurückhaltend interpretiert werden. Eine Stärke dieser Arbeit ist jedoch der modellbasierte Ansatz zum Vergleich der Behandlungssettings mittels Interaktionsanalysen.

Eine weitere Limitation ist die Möglichkeit von Confounding. Die Randomisierung aus der StärkeR-Hauptstudie, die nach Zentren stratifiziert war, ergab allerdings grundsätzlich vergleichbare Kollektive innerhalb der Zentren. Die deskriptiv beschriebenen, sich andeutenden Unterschiede zwischen den Behandlungsgruppen innerhalb des jeweiligen Settings sind rein zufällig entstanden und daher nicht durch Patientenpräferenzen, Krankheitszustand etc. beeinflusst worden. Der Vergleich der Behandlungsgruppen innerhalb der Settings ist damit zulässig und fair.

Da die Patient*innen aber nicht zufällig zu einem Behandlungssetting zugeordnet wurden, ist es möglich, dass Faktoren existieren, die beeinflussen, in welchem Behandlungssetting ein Patient vorstellig wird, und gleichzeitig einen Einfluss auf eines der interessierenden Outcomes haben. Es wäre z. B. denkbar, dass der Krankheitszustand der Patientin oder des Patienten ein solcher Faktor ist. Wenn diese Faktoren bei der Analyse nicht berücksichtigt werden, kann dies zu systematischen Verzerrungen bei der Schätzung der Interaktionseffekte führen. Die hier durchgeführten Analysen sind daher für potenzielle Confounder adjustiert worden, um solche Verzerrungen zu vermeiden. Allerdings könnten weitere Faktoren, die nicht erhoben wurden, wie z. B. die Anzahl an Verschreibungen für Physiotherapie oder das Ausmaß körperlicher Aktivität, und für die entsprechend nicht adjustiert werden konnte, die Ergebnisse verzerren.

Ebenso ist es möglich, dass die Umgebung und die Bedingungen in Klinikambulanzen sich von denen in Praxen unterscheiden, was sich auf die Wahrnehmung der Patient*innen auswirken könnte. Zum Beispiel könnten längere Wartezeiten in Klinikambulanzen zu einer geringeren Zufriedenheit der Patient*innen führen, obwohl die Behandlung selbst eigentlich gleichwertig war im Vergleich zur Behandlung in der Praxis. Solche „Messprobleme“, die durch unterschiedliche subjektive Wahrnehmung in den Settings verursacht werden, könnten ebenfalls zu verzerrten Ergebnissen führen.

Eine andere Erklärung für die beobachteten Unterschiede ist, dass es tatsächlich Unterschiede zwischen den Settings gibt. Grundsätzlich ist es denkbar, dass unterschiedliche Strukturen und Unterschiede bei den Behandelnden in Praxen und Klinikambulanzen die Umsetzbarkeit und damit auch den Erfolg eines teambasierten Behandlungskonzeptes beeinflussen. Die Ärzt*innen der Klinikambulanzen waren z. B. im Durchschnitt 10 Jahre jünger als die Niedergelassenen und damit im Mittel gleich alt wie die RFA. Fachliche Unterschiede bei den Behandelnden wären ebenso denkbar. Es ist möglich, dass die Ärzteschaft und auch die RFA in Klinikambulanzen und niedergelassenen Praxen unterschiedliche Herangehensweisen haben, die sich auf den Behandlungserfolg auswirken könnten. So kristallisierte sich in Fokusgruppen der StärkeR-Studie heraus, dass das Konzept der Delegation bzw. eine „RFA-Sprechstunde“ unterschiedlich implementiert wurde. Die RFA der Klinikambulanzen gaben übereinstimmend an, eine Versorgung mit Delegation passe aktuell nicht ins System, sodass die im Studienverlauf eingerichtete „RFA-Sprechstunde“ nach Beendigung der Studie nicht aufrechterhalten werden konnte. Die Möglichkeit des eigenverantwortlichen Arbeitens sei in den Klinikambulanzen auch nicht in der Form gegeben gewesen, wie sich das aus Berichten der niedergelassen tätigen RFA darstellte [6]. Dennoch waren sich die RFA beider Behandlungssettings einig, es führe kein Weg an der Delegation definierter Aufgaben an entsprechend qualifiziertes, nichtärztliches Personal vorbei, um die Belastung der Ärzt*innen zu reduzieren und so die Versorgung von Rheuma-Patient*innen zu verbessern.

Letztlich kann auf Grundlage der vorhandenen Daten nur vermutet werden, was die gefundenen Unterschiede zwischen den Behandlungssettings verursacht hat. Diese sollten unabhängig davon zusätzlich vor dem Hintergrund ihrer klinischen Relevanz hinterfragt werden. Ein zahlenmäßig deutlicherer Unterschied von ca. 7 % zwischen den Behandlungssettings deutet sich nur beim Endpunkt Funktionsfähigkeit an, die anderen, wenn auch signifikanten Unterschiede zwischen den Behandlungsgruppen im Praxissetting fallen hingegen eher gering aus.

Trotz ihrer methodischen Einschränkungen legt unsere Studie weitere Untersuchungen des Delegationskonzeptes vergleichend für Klinikambulanzen und Schwerpunktpraxen nahe, da eine teambasierte Versorgungsform mit Delegation an qualifizierte RFA eine notwendige und sinnvolle Ergänzung zur bisherigen Standardversorgung darstellt. Tiefer gehende Analysen könnten dabei wichtige Hinweise auf notwendige Voraussetzungen einer gelingenden Delegation an RFA liefern.

## Fazit

- Das Behandlungssetting könnte einen Einfluss auf die Ergebnisse einer teambasierten Versorgung mit Delegation an qualifizierte RFA haben.
- Die Determinanten der gefundenen Interaktion zwischen Versorgungsform und Behandlungssetting bleiben unklar und sollten weiter untersucht werden, um die notwendigen Voraussetzungen für eine effektive Delegation an RFA zu schaffen.

## Data Availability

Die Daten werden auf begründeten Antrag zur Verfügung gestellt.
